# Cytotoxic Effects of CdSe Quantum Dots on Maturation of Mouse Oocytes, Fertilization, and Fetal Development

**DOI:** 10.3390/ijms10052122

**Published:** 2009-05-14

**Authors:** Ming-Shu Hsieh, Nion-Heng Shiao, Wen-Hsiung Chan

**Affiliations:** Department of Bioscience Technology and Center for Nanotechnology, Chung Yuan Christian University, Chung Li, Taiwan

**Keywords:** quantum dot, apoptosis, oocyte maturation, embryonic development

## Abstract

Quantum dots (QDs) are useful novel luminescent markers, but their embryonic toxicity is yet to be fully established, particularly in oocyte maturation and sperm fertilization. Earlier experiments by our group show that CdSe-core QDs have cytotoxic effects on mouse blastocysts and are associated with defects in subsequent development. Here, we further investigate the influence of CdSe-core QDs on oocyte maturation, fertilization, and subsequent pre- and postimplantation development. CdSe-core QDs induced a significant reduction in the rates of oocyte maturation, fertilization, and *in vitro* embryo development, but not ZnS-coated CdSe QDs. Treatment of oocytes with 500 nM CdSe-core QDs during *in vitro* maturation (IVM) led to increased resorption of postimplantation embryos and decreased placental and fetal weights. To our knowledge, this is the first study to report the negative impact of CdSe-core QDs on mouse oocyte development. Moreover, surface modification of CdSe-core QDs with ZnS effectively prevented this cytotoxicity.

## 1. Introduction

Quantum dots (QDs), colloidal nanocrystalline semi-conductors with unique light emitting properties, are employed as novel luminescent materials. Typical QDs are 1 – 12 nm in diameter and contain a small number of atoms in any one lattice [1]. The lowest level QD energy absorption occurs at longer wavelengths and the energy is converted to narrow bandwidth emission. The particles are ideal for development as luminescent probes because of several advantageous characteristics, including broadband excitation, narrow bandwidth emission, high intensity of emitted light, good photostability, and photochemical stability [2–4]. These properties allow the effective application of QDs in biochemical assays, in particular, immunofluorescence staining. Previous studies show that CdSe-core QDs induce cell death [5,6] via an increase in reactive oxygen species (ROS), and inhibit survival-related signaling events, such as Ras and Raf-1 protein expression and extracellular regulated kinase (ERK) activation [6]. Additionally, a recent study by our group disclosed that CdSe-core QDs promote mouse embryo apoptosis and developmental injury of mouse blastocysts [7]. However, the effects of CdSe QDs on oocyte maturation and fertilization remain to be established.

Oocyte viability is affected by the microenvironment during growth and maturation. For instance, heat stress, oxygen concentration and glucose content are important determinants of oocyte viability [8–10]. The mycotoxin, citrinin, has hazardous effects on oocyte maturation, fertilization, and sequential embryonic development [11]. These results collectively imply that oocyte maturation is extremely sensitive to microenvironment changes and extracellular chemical compounds. A previous study reported that CdSe-core QDs induced apoptosis and embryonic developmental injury at the blastocyst stage [7]. Thus, CdSe QDs appear to exert latent cytotoxicity on the early stages of embryogenesis, such as oocyte maturation. During normal embryogenesis, apoptosis (a unique morphological pattern of cell death) functions to clear abnormal or redundant cells in preimplantation embryos [12,13]. However, apoptotic processes do not occur prior to the blastocyst stage during normal mouse embryonic development [14], and induction of cell death during oocyte maturation and early stages of embryogenesis (i.e., via exposure to a teratogen) leads to embryonic developmental injury [9,15,16]. CdSe-core QDs evidently promote apoptosis and developmental injury in blastocysts [17,18].

In our previous study, mouse blastocyst-stage embryos, developed from the zygote for four days, were directly treated with QDs for 24 h and we found that CdSe-core QDs induced mouse blastocyst apoptosis in a dose-dependent manner. Pretreatment of blastocysts with CdSe-core QDs inhibited cell proliferation, primarily in the inner cell mass. However, no studies have explored whether CdSe QDs adversely affect stages in early embryogenesis, such as oocyte maturation, fertilization, and subsequent embryo development. In this study, we further investigated the effect of oocyte treatment with CdSe-core QDs. The QDs were present for 24 h only, and the QD-treated oocytes were next incubated in QD-free medium to analyze the long-term effects of QDs on early stages in embryogenesis, including oocyte maturation, fertilization, and subsequent pre- and post-implantation development. Our results indicate that even short-term exposure of oocytes to CdSe-core QDs results in long-term toxic effects on embryogenesis, including oocyte maturation, fertilization, and subsequent embryonic development. Even though some zygotes derived from QD-treated oocytes were able to develop beyond the blastocyst stage, where uterine implantation occurs, we also found that such blastocysts had a higher level of apoptotic cells than had controls, resulting in a lower rate of successful embryogenesis. However, this cytotoxic effect was prevented by surface modification or coating with an appropriate shell, such as ZnS. Our results support the further development of ZnS-coated CdSe QDs as powerful fluorescent cellular tracers.

## 2. Results and Discussion

**Figure 1.**
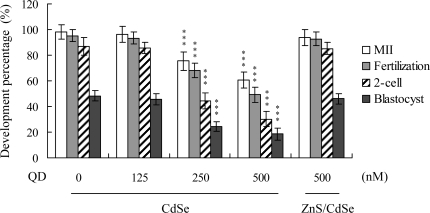
Effects of CdSe-core QDs on mouse oocyte maturation and embryo development *in vitro*.

While CdSe-core QDs evidently induce apoptosis and developmental injury in mouse blastocysts [7], their effects on oocyte maturation are currently unclear. Oocyte nuclear maturation status was measured using ten independent experimental replicates (200 – 250 oocytes per group). The number of oocytes that reached the metaphase II (MII) stage of maturation after *in vitro* maturation (IVM) ranged at about 98%. A lower maturation rate was estimated in the CdSe-core QDs-treated oocyte group, which was dose-dependent (Figure 1). For detecting fertilization, male pronucleus formation was assessed over a minimum of ten replicates (~ 200 oocytes/group). The ability of oocytes to be fertilized by fresh sperm, assessed on the basis of male pronucleus formation, was significantly decreased upon pre-treatment with CdSe-core QDs prior to IVM (Figure 1).

We further analyzed *in vitro* embryo development to the 2-cell and blastocyst stages. CdSe-core QDs pretreatment during IVM caused an injury effect, as evident from the decreased number of oocytes cleaved to the 2-cell stage (Figure 1). In addition, the number of embryos that cleaved and developed to form blastocysts in the CdSe-core QDs-treated groups were significantly lower than that in the untreated control groups (Figure 1).

Oocytes were collected from 21 day-old mice, cultured for 24 h in IVM medium with or without CdSe-core QDs (CdSe; 125, 250 or 500 nM) or ZnS-coated CdSe QDs (ZnS/CdSe; 500 nM), fertilized *in vitro*, and transferred to *in vitro* culture (IVC) medium. The levels of oocyte maturation, *in vitro* fertilization, cleavage, and blastocyst development were calculated with reference to total oocyte numbers. Values are presented as means ± SD of ten determinations. Data are based on 200-250 samples in each group. ***P < 0.001 versus the untreated control group.

**Figure 2.**
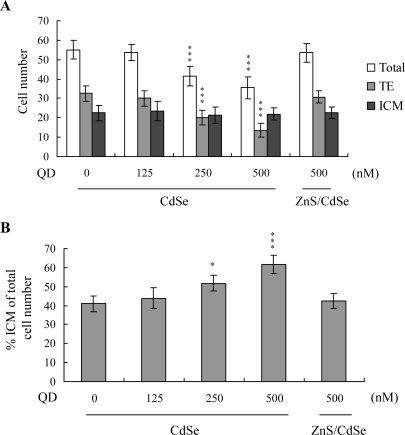
Effects of CdSe-core QDs during IVM of oocytes on cell number in embryos.

The total blastocyst cell number decreased following CdSe-core QDs treatment during *in vitro* maturation (IVM) of oocytes. Cell proliferation was assessed by differential staining, followed by cell counting. Significantly lower cell numbers of blastocysts were derived from CdSe-core QDs-pretreated oocytes, compared to those from control oocytes (Figure 2A). The number of trophectoderm (TE) cells in blastocysts also decreased during IVM upon pretreatment with CdSe-core QDs (Figure 2A). However, CdSe-core QDs application during IVM did not affect the number of inner cell mass (ICM) cells present in blastocysts (Figure 2A). The ICM-total cell ratio (expressed as a percentage) was higher in blastocysts derived from the CdSe-core QDs group during IVM, compared with untreated oocytes (Figure 2B).

Oocytes were cultured for 24 h in IVM medium with or without CdSe-core QDs (CdSe; 125, 250 or 500 nM) or ZnS-coated CdSe QDs (ZnS/CdSe; 500 nM), fertilized *in vitro*, and transferred to *in vitro* culture (IVC) medium for *in vitro* development. (A) Total cell count in blastocysts, trophectoderm (TE) lineages and inner cell mass (ICM). (B) The percentage of ICM cells in relation to total cell number was analyzed. Data is based on at least 320 samples per group. *P < 0.05 and ***P < 0.001 versus the untreated control group.

**Figure 3.**
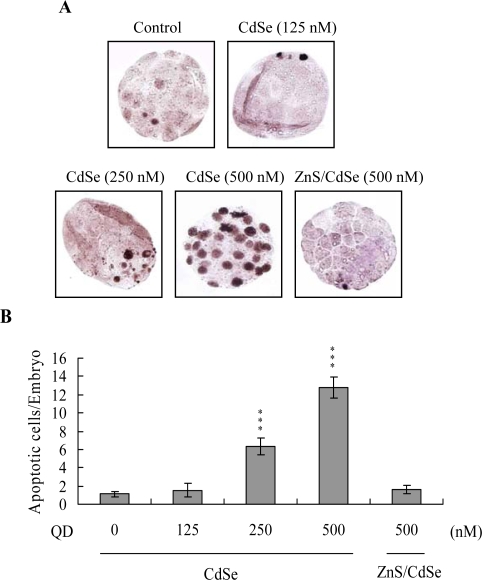
Effects of CdSe-core QDs administered during IVM of oocytes on apoptosis in embryos. (A) Oocytes were cultured for 24 h in IVM medium with or without CdSe-core QDs (CdSe; 125, 250 or 500 nM) or ZnS-coated CdSe QDs (ZnS/CdSe; 500 nM), fertilized *in vitro*, and transferred to *in vitro* culture (IVC) medium for development. Apoptotic cells were examined by TUNEL staining, followed by light microscopy (positive cells are depicted in black). (B) The mean number of apoptotic (TUNEL-positive) cells per blastocyst was calculated. Values are presented as means ± SD of eight determinations. Data are based on at least 250 samples in each group. ***P < 0.001 versus the untreated control group.

Apoptosis of blastocysts derived from CdSe-core QDs-pretreated oocytes was additionally evaluated. Cell death type was analyzed by TUNEL staining; this is the recognized assay for apoptosis. TUNEL staining revealed enhanced dose-dependent blastocyst apoptosis in the CdSe-core QDs-pretreated oocyte group, (Figure 3A; apoptotic cells are shown in black). Quantitative analysis disclosed a 6- (250 nM QDs) to 13-fold (500 nM QDs) increase in apoptotic blastocysts derived from CdSe-core QDs-pretreated oocytes compared to the control group (Figure 3B). Moreover, according to the data shown in Figures 2 and 3, we suggest that apoptotic cells of blastocysts derived from CdSe-core QDs-treated oocytes are prominent in TE cells.

**Figure 4.**
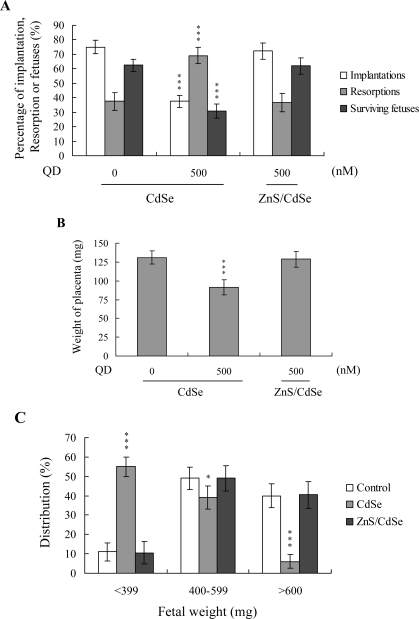
Effects of CdSe-core QDs treatment during oocyte IVM on embryonic *in vivo* implantation, resorption, fetal survival and fetal weight. Oocytes were cultured for 24 h in IVM medium with or without CdSe-core QDs (CdSe; 500 nM) or ZnS-coated CdSe QDs (ZnS/CdSe), fertilized *in vitro*, and transferred to *in vitro* culture medium for development. (A) Implantation, resorption, and surviving fetuses were analyzed, as described in Materials and Methods. The implantation percentage represents the number of implantations per number of transferred embryos × 100. The number of resorptions or surviving fetuses per number of implantations is expressed as a percentage (× 100). (B) Placental weights of 40 recipient mice were measured. (C) Weight distribution of surviving fetuses on day 18 post-coitus. Surviving fetuses were obtained by embryo transfer of control, CdSe-core QDs-pretreated groups or ZnS-coated CdSe QDs-pretreated groups, as described in Materials and Methods (320 total blastocysts across 40 recipients). ***P < 0.001 versus the CdSe-core QDs-free group.

Embryos were transferred to 55 recipients (eight per horn). A total of 40 recipients were pregnant in at least one horn at day 18. The implantation ratio of blastocysts derived from the CdSe-core QDs-treated oocyte group during IVM was ~ 37.5% (120 of 320 embryos in 40 recipients), which was significantly lower than that estimated for control blastocysts (~ 75%; 240 of 320 embryos in 40 recipients) (Figure 4A). In addition, the proportion of implanted embryos that failed to develop normally was significantly higher in the CdSe-core QDs-treated group (83 of 120 implanted embryos; ~ 69%) than the control group (90 of 240 implanted embryos; ~ 37.5%). Embryos that implanted but failed to develop were subsequently resorbed in the uterus. The group pre-treated with CdSe-core QDs displayed a higher resorption rate than the untreated control group (Figure 4A). With regard to the embryo survival rate, 62.5% (150 of 240 implanted embryos) of the control group and only 30.8% (37 of 120 implanted embryos) of the CdSe-core QDs-treated group survived at post-coitus day 18 (Figure 4A).

Moreover, the placental weights of blastocysts derived from CdSe-core QDs-treated oocytes in IVM were significantly lower than those in the control group (Figure 4B). Fetal weights were lower in the CdSe-core QDs-treated group, relative to the control group (485 ± 62 mg versus 615 ± 58 mg). Only 6% of fetuses in the pretreated group weighed over 600 mg, whereas 40% of control fetuses exceeded this threshold, an important indicator of successful embryonic and fetal development (Figure 4C). Our findings collectively indicate that exposure of oocytes to CdSe-core QDs in IVM reduces the potential of postimplantation development.

Next, we investigated the capacity of a ZnS coating to reduce CdSe QDs-induced cytotoxic effects on oocyte maturation and embryonic development. Interestingly, ZnS-coated CdSe QDs had no significant cytotoxic effects on oocyte maturation status, fertilization rate or *in vitro* embryo development (Figure 1). Moreover, cell proliferation and TUNEL staining analyses revealed no significant injury effects of ZnS-coated CdSe QDs on blastocysts during IVM of oocytes (Figures 2 and 3). Accordingly, we conclude that ZnS coating of CdSe QDs suppresses the observed cytotoxic effects on oocyte maturation, fertilization rate and sequential embryonic development.

The induction of cell death by CdSe-core QDs under specific conditions correlates with the release of free Cd^2+^ from the CdSe lattice. This effect is significantly reduced by coating QDs with ZnS [5]. Photoluminescent semiconductor QDs are novel nanometer-size fluorescent probes used for the bioimaging of immunostained cells [19], and are valuable tools for tracing target cells over time in mouse models [20]. Fluorescent QD probes can be potentially developed as biotracers for human disease diagnosis. Thus, it is essential to clarify the cytotoxic capacity of QDs. A previous study by our group demonstrates that CdSe-core QDs-induced apoptosis and inhibition of embryonic development is effectively reduced with a ZnS coat [7]. These results suggest that QDs could exert latent cytotoxicity in the event of disruption of the ZnS coat *in vivo*. In this report, we further investigate the cytotoxic effects of CdSe QDs on oocyte maturation, fertilization, and embryo development during IVM. Treatment of oocytes with CdSe QDs led to injury effects on maturation, fertilization, and embryonic development (Figures 1 and 4). In our experience, CdSe QD levels of about 300–500 nM show potent fluorescence intensities and are suitable for cell staining analysis by fluorescence microscopy. In addition, earlier studies used the CdSe QD concentrations employed for *in vitro* cell staining or in animal labeling studies [5,20–22]. Together, these results imply that the concentration of CdSe QDs we employed (250 – 500 nM) has latent cytotoxicity, after the surface protection shells are destroyed.

Cadmium (Cd) is a significant environmental and occupational toxic metal. Cd exerts pro- or anti-apoptotic effects, depending on the cell type, dosage and exposure period. The metal induces apoptosis in T lymphocytes [23], LLC-PK1 cells [24], canine proximal tubules [25], and rat testicular tissue [26]. Apoptosis is triggered via both caspase-dependent [27] and -independent [28] pathways. Cd additionally blocks apoptosis stimulated by a variety of agents [29]. Other studies show that Cd activates Ca^2+^/calmodulin-dependent protein kinase II in mouse mesangial cells, and triggers apoptotic and necrotic cell death [30]. Cd also induces ROS generation (i.e., oxidative stress) and apoptosis via a caspase-dependent pathway [31]. However, the mechanisms underlying Cd-induced apoptosis, cell cytotoxicity and carcinogenesis are unclear at present.

Selenium (Se) is an essential dietary trace element for humans. There are at least eight Se-containing proteins, including glutathione peroxidase and thioredoxin reductase. Dietary Se supplementation can protect cells from oxidative injury [32] and inhibits apoptosis [33,34]. Conversely, Se can also trigger apoptosis [34,35]. Further investigation is required to unravel the mechanisms underlying the protective and apoptotic effects of selenium.

The TE arises from the trophoblast at the blastocyst stage, and develops into a sphere of epithelial cells surrounding the ICM and blastocoel. These cells contribute to the placenta, and are required for mammalian conceptus development [36], indicating that a reduction in the TE cell lineage leads to suppressed implantation and embryonic viability [37,38]. Based on the finding that mouse blastocysts derived from oocytes treated with CdSe-core QDs during IVM display reduced TE cell numbers, we investigated the possibility that CdSe-core QDs cause mortality and/or developmental delay in postimplantation mouse embryos. Our results further confirm that blastocysts derived from CdSe-core QDs-treated oocytes undergo decreased implantation, increased embryo resorption, and decreased fetus survival rate (Figure 4A). In addition, CdSe-core QDs induced a significant reduction in placental and fetal weights, compared to the untreated control group (Figures 4B,C). TE cells of embryos play important roles in implantation and placental development. Accordingly, we propose that the decrease in TE cell number by CdSe-core QDs during oocyte maturation is a major injury factor in embryonic development.

We previously showed that co-incubation of CdSe-core QDs with mouse blastocyst stage embryos for 24 h could trigger cell apoptosis, inhibit cell proliferation, retard early postimplantation blastocyst development, and increase early-stage blastocyst death both *in vitro* and *in vivo* [7]. In the present work, we directly incubated mouse oocytes with CdSe QDs for 24 h and analyzed the effects of such incubation, using a CdSe QD-free incubation system, on oocyte maturation status, fertilization, and embryogenesis. Importantly, we found that CdSe QDs induced a significant reduction in all of these developmental features. Interestingly, the detrimental effect of CdSe-core QDs on oocytes was long-term, because blastocyst-stage embryos (that developed from zygotes in about 4 days) arising from CdSe-core QDs-treated oocytes under QDs-free maturation conditions showed a higher apoptotic cell rate than did untreated controls. This implies that uptake of CdSe-core QDs by oocytes results in long-term QD accumulation in subsequent cells of various embryonic stages arising by mitosis from zygotes, and negatively affects different stages of embryogenesis. The results also show that exposure to CdSe-core QDs for even a short period of oocyte life potently affects subsequent steps in embryogenesis, in a negative manner.

Previous work showed that CdSe QD cytotoxicity correlated with free Cd^2+^ release from the CdSe lattice, which appeared to be associated with surface oxidation [5]. Recently, in a preliminary study, we further found that incubation of CdSe-core QDs (500 nM) in oocyte IVM medium for 24 h yielded 17.5 – 18.4 ppm free medium Cd^2+^, as observed by inductively coupled plasma optical emission spectroscopy (ICP/OES) analysis. In addition, earlier studies demonstrate that the addition of a ZnS coating to CdSe QDs significantly reduces associated cytotoxicity [5,7]. Accordingly, the effects of ZnS on CdSe-core QDs cytotoxicity during oocyte maturation were investigated. Our data show that a ZnS coating effectively lowers CdSe QDs-induced cytotoxicity in oocyte maturation, fertilization, and sequential embryonic development (Figures 1–3). We propose that the ZnS coating prevents cytotoxicity by blocking surface oxidation and the subsequent release of Cd^2+^ ions.

## 3. Experimental Section

### 3.1. Chemicals and Reagents

Dulbecco’s modified Eagle’s medium (DMEM) and pregnant mare serum gonadotropin (PMSG) were obtained from Sigma (St. Louis, MO, USA). Human chorionic gonadotropin (hCG) was purchased from Serono (NV Organon Oss, The Netherlands). TUNEL *in situ* cell death detection kits were acquired from Roche (Mannheim, Germany), and CMRL-1066 medium from Gibco Life Technologies (Grand Island, NY, USA).

### 3.2. Quantum dot preparation

Nanocrystals comprising a CdSe core and a ZnS shell were synthesized. Briefly, appropriate amounts of trioctylphosphine oxide (TOPO), cadmium oxide (CdO) and tetradecylphosphonic acid (TDPA) were heated to 180 °C under argon, and dried and degassed under vacuum. The reaction temperature was then increased to 330 °C, selenium (Se) precursor solution in trioctylphosphine (TOP) was injected into the reaction flask, and the mixture was allowed to cool to 240 °C. Zn and S stock solutions prepared with bis(trimethylsilyl)sulfide in TOP, along with a dimethyl zinc solution, were added dropwise with vigorous stirring until a final mole ratio of 1:4 (Cd/Se:Zn/S) was achieved in the reaction. The reaction mixture was cooled to room temperature, and the nanocrystals were precipitated with anhydrous methanol, collected by centrifugation, and washed three times with anhydrous methanol for removal of residual TOPO and unreacted reagents. The precipitate was dissolved in anhydrous chloroform or tetrahydrofuran (THF) for experiments. For water solubilization, the CdSe QDs were surface coupled with mercaptoacetic acid (MAA) and then suspended in PBS buffer. A particle sizer was used to measure the CdSe QDs, which were found to be about 3.5 nm in diameter.

### 3.3. COC collection and in vitro maturation (IVM)

ICR mice were acquired from the National Laboratory Animal Center (Taiwan, ROC). This research was approved by the Animal Research Ethics Board of Chung Yuan Christian University (Taiwan, ROC). All animals received humane care, as outlined in the Guidelines for Care and Use of Experimental Animals (Canadian Council on Animal Care, Ottawa, 1984). Mice were maintained on breeder chow (Harlan Teklad chow) with food and water available *ad libitum*. Housing was provided in standard 28 cm × 16 cm × 11 cm (height) polypropylene cages with wire-grid tops, and maintained under a 12 h day/12 h night regimen. Cumulus-oocyte complexes (COCs) were obtained according to a previous protocol [9]. Briefly, COCs were isolated from female hybrid ICR mice (21 days old) injected with 5 IU human chorionic gonadotrophin (hCG) 44 h prior to oocyte collection. COCs were collected in HEPES-buffered α minimum essential medium (MEM) (containing 50 μg/mL Streptomycin sulfate, 75 μg/mL Penicillin G, and 5% fetal bovine serum) by gently puncturing visible antral follicles present on the ovary surface. Germinal vesicle stage oocytes containing an intact vestment of cumulus cells were collected and pooled in at least 8 animals. For oocyte maturation, one drop (~ 100 μL) of buffer (αMEM supplemented with 50 μg/mL Streptomycin, 75 μg/mL Penicillin G, 5% FBS and 50 mIU/mL recombinant human FSH) containing 10 COCs was added under oil in 35 mm culture dishes. COC maturation was analyzed following treatment with or without various concentrations of CdSe QDs (0–500 nM) for 24 h under an atmosphere of 5% O_2_, 6% CO_2_ and balance of N_2_ at 37 °C.

### 3.4. Maturation Status assessment

After *in vitro* maturation (IVM), COCs of each group were treated with 50 U/mL ovine hyaluronidase and gently pipetted for the removal of all cumulus cells. Denuded oocytes were collected, and washed with fresh medium, followed by phosphate-buffered saline (PBS). Oocytes were fixed in ethanol: glacial acetic acid (1:3) for 48 h, and stained with 1% aceto-orcein solution. Nuclear structures were visualized using phase-contrast microscopy.

### 3.5. In vivo maturation

For obtaining *in vivo* matured oocytes, 21 day-old mice were injected with 5 IU equine chorionic gonadotrophin (eCG) and 5 IU hCG, 61 and 13 h prior to fertilization, respectively. Mature ova were collected from the oviduct into HEPES-buffered α-MEM medium.

### 3.6. In vitro fertilization

For *in vitro* fertilization, ova were washed twice in bicarbonate-buffered α-MEM medium (containing 50 mg/mL streptomycin, 75 mg/mL penicillin G and 3 mg/mL fatty acid free bovine serum albumin), and fertilized in the same medium with fresh sperm (obtained from a CBAB6F1 male donor). After incubation with sperm for 4.5 h, eggs were washed three times in potassium simplex optimized medium (KSOM) without amino acids in the presence of l-alanyl-l-glutamine (1.0 mM). Next, eggs were placed in 20 mL drops of KSOM under oil, and cultured overnight. During cleavage to the 2-cell stage, embryos were transferred to a fresh drop of KSOM under oil, and cultured for another 72 h. All fertilization steps and embryo culture were additionally carried out under 5% O_2_, 6% CO_2_ and balance of N_2_ at 37 °C.

### 3.7. Fertilization assessment

For the examination of fertilization, ova were incubated with sperm for 4.5 h, followed by 3 h of culture in fresh medium. Zygotes were assessed for the presence of the male pronucleus with orcein staining, as described previously [9].

### 3.8. Cell Proliferation

Cell proliferation was analyzed by dual differential staining, which facilitated the counting of cell numbers in inner cell mass (ICM) and trophectoderm (TE) [37]. Blastocysts were incubated with 0.4% pronase in M_2_-BSA medium (M_2_ medium containing 0.1% bovine serum albumin) for the removal of zona pellucida. Denuded blastocysts were exposed to 1 mM trinitrobenzenesulfonic acid (TNBS) in BSA-free M_2_ medium containing 0.1% polyvinylpyrrolidone (PVP) at 4 °C for 30 min, and washed with M_2_ [39]. Blastocysts were further treated with 30 μg/mL anti-dinitrophenol-BSA complex antibody in M_2_-BSA at 37 °C for 30 min, followed by M_2_ supplemented with 10% whole guinea pig serum as a source of complement, along with 20 μg/mL bisbenzimide and 10 μg/mL propidium iodide (PI) at 37 °C for 30 min. The immunolysed blastocysts were gently transferred to slides, and protected from light before observation. Under UV light, ICM cells (which take up bisbenzimidine but exclude PI) appeared blue, whereas TE cells (which take up both fluorochromes) appeared orange-red. Since multinucleated cells are not common in preimplantation embryos [40], the number of nuclei represent an accurate measurement of cell number.

### 3.9. TUNEL assay of blastocysts

For TUNEL staining, embryos were washed in QDs-free medium, fixed, permeabilized, and subjected to labeling using an *in situ* cell death detection kit (Roche Molecular Biochemicals, Mannheim, Germany), according to the manufacturer’s protocol. Photographic images were obtained with a fluorescence microscope under bright-field illumination.

### 3.10. Blastocyst development following embryo transfer

To determine the ability of expanded blastocysts to implant and develop *in vivo*, embryos generated were transferred to recipient mice. ICR females (6–8 week-old, white skin) were mated with vasectomized males (C57BL/6J; black skin; National Laboratory Animal Center, Taiwan, ROC) to produce pseudopregnant dams as recipients for embryo transfer. To ensure that all fetuses in pseudopregnant mice were derived from embryo transfer (white color) and not fertilization by C57BL/6J (black color), we examined skin color at day 18 post-coitus. To assess the impact of CdSe QDs on postimplantation growth *in vivo*, COCs were exposed to 0 and 500 nM CdSe-core QDs for 24 h, followed by fertilization and *in vitro* maturation to the blastocyst stage. Subsequently, eight untreated control embryos were transferred to the left uterine horn, and eight CdSe-core QDs-treated embryos to the right uterine horn in day four pseudopregnant mice. Forty surrogate mice were analyzed and killed on day 18 post-coitus, and the frequency of implantation calculated as the number of implantation sites per number of embryos transferred. The incidence rates of resorbed and surviving fetuses were calculated as number of fetuses per number of implantations, respectively. The weights of the surviving fetuses and placentae were measured immediately after dissection.

### 3.11. Statistical Analysis

Data were analyzed using one-way ANOVA and t-tests, and presented as means ± SD. Data were considered statistically significant at *P* < 0.05.

## 4. Conclusions

In sum, this report demonstrate that exposure of oocytes to CdSe-core QDs during IVM decreases cell number, induces apoptosis, and inhibits postimplantation development, possibly due to a teratogenic effect. Moreover, these hazardous effects of CdSe-core QDs could be avoided by surface coating with ZnS. Evidently, CdSe-core QDs have a teratogenic effect on oocyte maturation and embryo development, and appropriate surface modifications may effectively block this toxicity. These findings should facilitate the development of ZnS-coated CdSe QDs as a powerful fluorescence tracer for oocyte or embryonic maturation analysis.
